# Longitudinal trends in physical activity and sleep before, during, and after pregnancy using Fitbit and EHR data from the *All of Us* research program

**DOI:** 10.1016/j.ajogmf.2025.101744

**Published:** 2025-07-18

**Authors:** Claire Lo, Jeffrey Annis, Hiral Master, Adnan Cakar, Sarah Osmundson, Douglas Ruderfer, Evan Brittain

**Affiliations:** Department of Medicine, Vanderbilt University Medical Center, Nashville, TN (Lo); Vanderbilt Institute of Clinical and Translational Research, Vanderbilt University Medical Center, Nashville, TN (Annis, Master, and Cakar); Department of Obstetrics and Gynecology, Vanderbilt University Medical Center, Nashville, TN (Osmundson); Division of Genetic Medicine, Vanderbilt Genetics Institute, Vanderbilt University Medical Center, Nashville, TN (Ruderfer); Center for Digital Genomic Medicine, Department of Medicine, Vanderbilt University Medical Center, Nashville, TN (Ruderfer and Brittain); Division of Cardiovascular Medicine, Vanderbilt University Medical Center, Nashville, TN (Brittain).

## Objectives

Understanding how physical activity and sleep change during pregnancy and postpartum is essential for supporting maternal health. Prior research has relied on self-report or short-term monitoring and generally has excluded the preconception period.^1–3^ This study leveraged longitudinal commercial wearable data from the National Institutes of Health (NIH) *All of Us* Research Program (AoURP) to describe within-person changes in daily step counts and sleep duration before, during, and after pregnancy.

## Study design

Live births (2018–2022) were extracted using the *All of Us* Research Workbench among participants aged 18 to 45 at delivery. Pregnancies were identified via CPT codes for vaginal or cesarean delivery and matched 3:1 to controls. Pregnancy start dates were estimated by subtracting 280 days from delivery dates (see Supplement).

Commercial wearable data were obtained from Fitbit devices. Inclusion required linked Fitbit data with ≥10 hours/day wear time, monitoring within 365 days pre- and postdelivery, and ≥90 days of data both before and after delivery. Outliers were excluded based on implausible values (ie, <100 or >45,000 steps/day; sleep >24 or <0 hours; sleep >30% of days with <4 hours sleep). Daily activity and sleep metrics were modeled using generalized least squares accounting for nonlinear interactions between time, pregnancy, and postpartum status. Recovery was defined as the first day predicted step count differences were no longer significant (*P*<.05) and confirmed by convergence of predicted means. Additional analytic details are provided in the Supplement.

## Results

We included 51 and 24 pregnant participants in the activity and sleep analyses, respectively (Supplemental Figure 1.1, 1.2). Participants were predominantly White (>58% White; <20% non-Hispanic Black, <20% other/multiple races) and geographically distributed across all U.S. Census regions (45% Midwest, 33% Northeast, <20% South and West).

**Figure 1:**
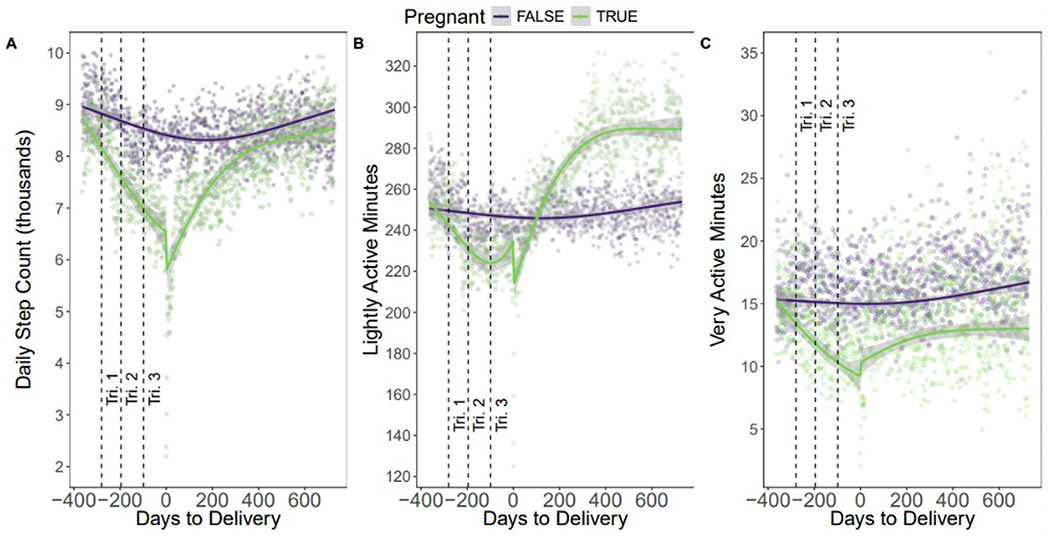
Weekly mean step counts aligned to delivery week. A, Weekly mean daily step counts from 60 weeks before to 80 weeks after delivery, with 95% confidence intervals. Step counts declined progressively throughout pregnancy, reaching a nadir in week 39, followed by partial recovery postpartum. B, Light intensity physical activity minutes from 60 weeks before to 80 weeks after delivery, with 95% confidence intervals. C, High intensity physical activity minutes from 60 weeks before to 80 weeks after delivery, with 95% confidence intervals.

Recovery to nonpregnant step levels occurred at a mean of 591 days postpartum (330 steps/day; 95% CI: [0, 662]; *P*=.051) (Figure 1, A). Lightly active minutes decreased during pregnancy (−19.8 minutes; 95% CI: [−30.1, −8.8]; *P*<.001), then rose sharply postpartum, surpassing both prepregnancy and control levels from about 90 days to 2 years postpartum (Figure 1, B). High-intensity minutes declined throughout pregnancy and remained below baseline postpartum (Figure 1, C), consistent with prior wearable-based findings.^2,4^

## Sleep duration

**Figure 2:**
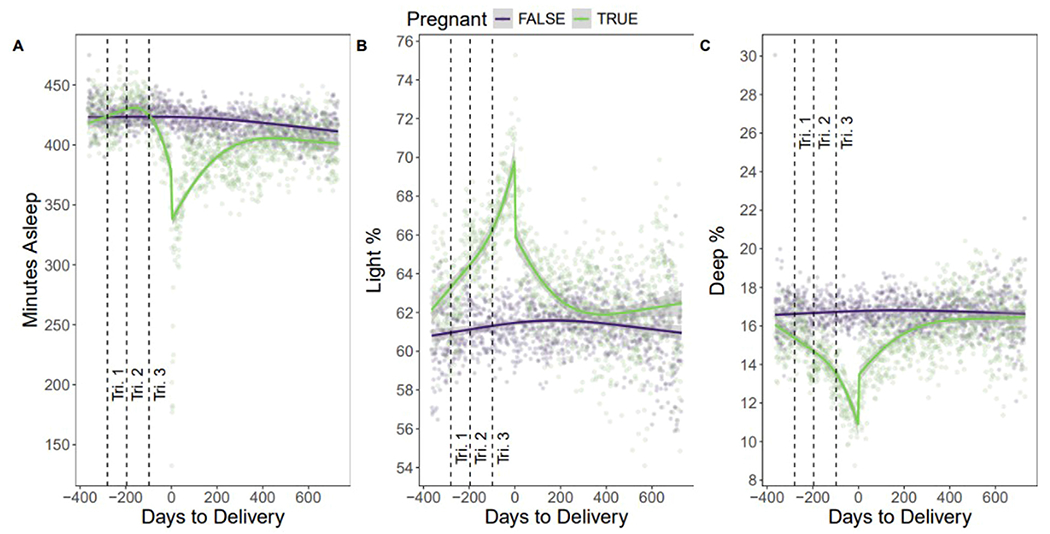
Weekly mean sleep metrics aligned to delivery week. A, Weekly mean nightly sleep duration (hours), showing a modest rise in early pregnancy and a sharp decline in the immediate postpartum period. Sleep duration did not fully return to baseline even by 1 year postpartum. B, Weekly mean percentage of time in light sleep, showing an increase in light sleep throughout pregnancy and gradual decrease no near baseline noted at 1-year postpartum. C, Weekly mean percentage of time in deep sleep, showing steady decrease in deep sleep throughout pregnancy and gradual recovery in deep sleep noted by 1-year postpartum.

Sleep duration declined in the third trimester, with near full recovery by 437 days postpartum (−12 minutes; 95% CI: [−18.0, −6.1]; *P*<.001) (Figure 2, A). Although time awake increased with pregnancy progression, this trend was not significant. Light sleep increased during pregnancy (Δ+6.5%; 95% CI: [5.5, 7.6]; *P*<.001), returning to baseline by 1 year postpartum (Figure 2, B), while deep sleep showed an opposing decline (Δ −4.5%; 95% CI: [−5.1, −3.9]; *P*<.001) (Figure 2, C). These trends align with prior actigraphy-based studies showing disrupted sleep architecture during pregnancy.^1,3,5^

Additional results are provided in the Supplement, including characterization of recovery based on convergence of predicted means.

## Delivery subtype

In an exploratory sub-analysis by delivery type, individuals who underwent cesarean section took fewer average daily steps prior to pregnancy (−365 days from delivery) (−900 steps/day CI: [−1550, 250], *P*=.007), during the first trimester (−280 to −187 days from delivery) (−1020 CI: [−1400, −650], *P*<.001), and at the end of the third trimester (−94 to −1 day from delivery) (−1240 CI: [−2210, −270], *P*=.012) compared to individuals who underwent vaginal delivery with larger differences observed between the cesarean and control groups.

## Conclusion

Leveraging longitudinal Fitbit and EHR data from a large cohort, we observed consistent declines in physical activity and disrupted sleep during pregnancy, followed by incomplete postpartum recovery—patterns with potential implications for maternal health. Step counts declined steadily through pregnancy, with a sustained increase in light intensity activity postpartum. Sleep duration followed a less linear trajectory, increasing modestly in early pregnancy and falling sharply postpartum, with incomplete recovery by 1 year.

These findings extend prior research by capturing the preconception period and providing high-resolution, within-person data over more than 2 years.^3^ Persistent postpartum reductions in activity and sleep underscore the need for support beyond the standard 6-week postpartum visit. This study demonstrates the value of integrating commercial wearable data with EHRs to monitor behavioral changes during major life transitions. As wearable adoption grows and data quality improves, digital phenotyping may enable early identification of individuals at risk for adverse postpartum outcomes and inform targeted interventions.

## Supplement

### Phenotyping cases and controls

Pregnancy cases were defined by the presence of at least one CPT code for vaginal or cesarean delivery in linked EHR data, marking the pregnancy end date. Controls lacked such codes during the matching period. Controls were matched 3:1 to cases based on age, prepregnancy BMI, baseline average daily step count or sleep duration, and monitoring period (start and end dates) to account for seasonal variation. Baseline was defined as the first 90 days of monitoring within 365 days before delivery, entirely preceding pregnancy onset.

### Demographic and clinical characteristics

Demographic characteristics were derived from AoURP participant surveys and EHRs. Race and ethnicity were self-reported and categorized as non-Hispanic White, non-Hispanic Black, Hispanic/Latino, Asian, and other/multiple races. BMI, parity, comorbidities and enrollment timing were extracted from EHRs.

### Fitbit data

Physical activity was assessed using Fitbit-derived daily summaries, including step counts and time spent in sedentary, lightly active, fairly active, and very active states. Per Fitbit algorithms, activity intensity was quantified using mean bout cadence, calculated by averaging the steps during which a participant engaged in ≥2 consecutive minutes at ≥60 steps/minute.

Sleep metrics included total sleep duration and sleep stages (ie, light, deep, REM), estimated by Fitbit’s proprietary algorithm for sleep periods >3 hours using heart rate and movement data.

### Statistical analyses

Matching was performed using nearest neighbor matching via Mahalanobis distance, based on age, sex (female), prepregnancy BMI, baseline Fitbit data (first 90 days), and monitoring period. Controls had no CPT codes indicating pregnancy. The event date was defined as the delivery date.

Statistical definitions of recovery are described in primary text. Time-to-event was modeled using restricted cubic splines with three knots. Models were adjusted for age and prepregnancy BMI and assumed a within-person autocorrelation structure of order 1. Exploratory analyses examined associations between Fitbit metrics and delivery type, as well as selected pregnancy and postpartum complications. Analyses were conducted in R within the *All of Us* Researcher Workbench.

### Wearable data inclusion and processing

All analyses were conducted in a secure, cloud-based environment and followed *All of Us* policies for data use and disclosure. Institutional Review Board approval was obtained through the *All of Us* single IRB. Fitbit data were extracted from the *All of Us* Curated Data Repository, which includes participant-provided, device-generated time series data synced from commercial devices.

### Additional results

We compared two analytic strategies to determine time to recovery: *P*-values versus convergence of predicted means. Recovery time typically different only by a few days when comparing *P*-values with convergence strategy. Time to recovery in step count differed the most between the two analytic strategies (288 days with convergence of predicted means; 330 days with *P*-values).

## Supplementary Material

1

Supplementary material associated with this article can be found in the online version at doi:10.1016/j.ajogmf.2025.101744.
