# Supplementary figures and images for: Longitudinal trends in physical activity and sleep before, during, and after pregnancy using Fitbit and EHR data from the All of Us research program
Source: Am J Obstet Gynecol MFM. Author manuscript; Available in PMC 2026 Jun 1. (PMC13224825; doi:10.1016/j.ajogmf.2025.101744)

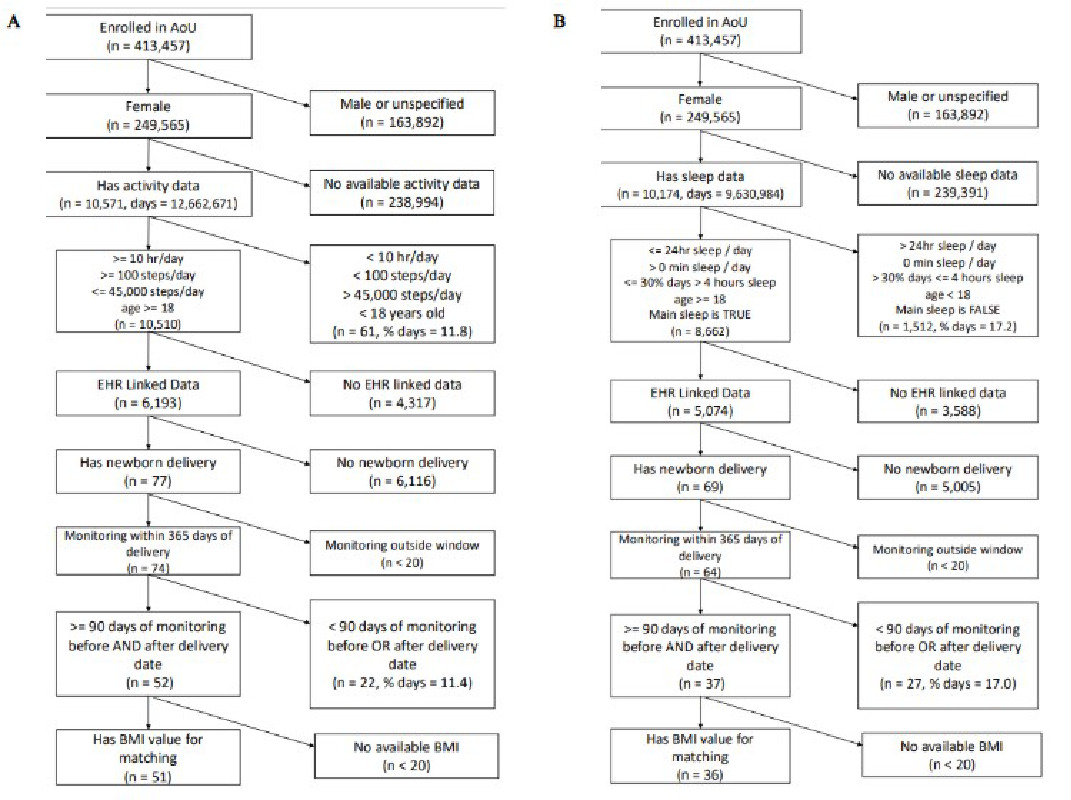

Supplement: 1 [file NIHMS2171293-supplement-1.jpg]
